# A Fast and Reliable UHPLC–MS/MS-Based Method for Screening Selected Pharmacologically Significant Natural Plant Indole Alkaloids

**DOI:** 10.3390/molecules25143274

**Published:** 2020-07-18

**Authors:** Danuše Tarkowská

**Affiliations:** Laboratory of Growth Regulators, Centre of the Region Haná for Biotechnological and Agricultural Research, Institute of Experimental Botany, Czech Academy of Sciences and Faculty of Science, Palacký University, CZ-78371 Olomouc, Czech Republic; tarkowska@ueb.cas.cz; Tel.: +420-585-631-478

**Keywords:** monoterpene indole alkaloids, monoamine oxidase inhibitors, harmaline, harmine, yohimbine, ajmalicine, plants, analysis, ultra-high performance liquid chromatography, mass spectrometry

## Abstract

Many substances of secondary plant metabolism have often attracted the attention of scientists and the public because they have certain beneficial effects on human health, although the reason for their biosynthesis in the plant remains unclear. This is also the case for alkaloids. More than 200 years have passed since the discovery of the first alkaloid (morphine), and several thousand substances of this character have been isolated since then. Most often, alkaloid-rich plants are part of folk medicine with centuries-old traditions. What is particularly important to monitor for these herbal products is the spectrum and concentrations of the present active substances, which decide whether the product has a beneficial or toxic effect on human health. In this work, we present a fast, reliable, and robust method for the extraction, preconcentration, and determination of four selected alkaloids with an indole skeleton, i.e., harmine, harmaline, yohimbine, and ajmalicine, by ultra-high performance liquid chromatography coupled with tandem mass spectrometry. The applicability of the method was demonstrated for tobacco and *Tribulus terrestris* plant tissue, the seeds of *Peganum harmala,* and extract from the bark of the African tree *Pausinystalia johimbe*.

## 1. Introduction

**Figure 1:**
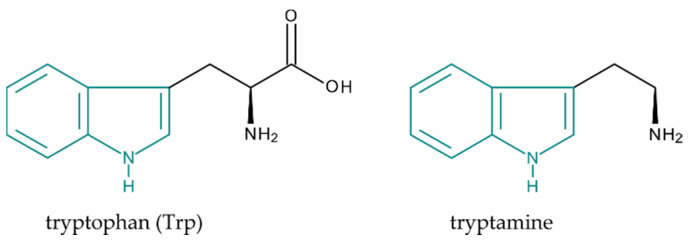
Structure of tryptophan (Trp) and tryptamine with indole ring marked in teal.

Alkaloids are a well-known and important group of natural substances. The name “alkaloid” is derived from the alkaline nature of most of these substances, thanks to which it forms salts with acids. Most alkaloids occur in flowering plants as products of secondary metabolism, mainly in the form of salts with organic acids (acetic, oxalic, citric, lactic, tartaric), rarely in free form [1]. Alkaloids occur most often in the higher dicotyledon plants, and, to a lesser extent, in monocotyledons, e.g., the Liliaceae family. Even some seedless plants contain alkaloids, e.g., the yew tree (*Taxus*), joint pine (*Ephedra*), ground pine (*Lycopodium*), or horsetail (*Equisetum*). However, alkaloids are also present in some fungi, such as *Claviceps purpurea* or *Psilocybe*. The structure of alkaloids is very diverse: they can be aliphatic, cyclic, and aromatic, and they can also often have a steroidal structure. Biogenetically, alkaloids are mostly derived from amino acids, namely, ornithine, lysine, phenylalanine, tyrosine, tryptophan, and histidine. The subject of this article is indole alkaloids (IAs) in particular. This group of alkaloids have a common structural feature, the indole skeleton, which is biosynthetically derived from amino acid tryptophan (Trp; Figure 1). Most IAs also contain isoprenoid building blocks being formed via both known isoprenoid pathways—the mevalonate pathway (MVA) and the nonmevalonate pathway, also called the 2-*C*-methyl-d-erythritol 4-phosphate pathway (MEP) [2]. According to the structure of the terpene unit, we distinguish simple IAs (including one C_5_ unit—ergot alkaloids) and terpene IAs (including two C_5_ units and containing 9–10 carbons: *Catharanthus*, *Rauvolfia,* and *Strychnos* alkaloids). Simple IAs are derivatives of tryptamine (Figure 1), which is a decarboxylation product of Trp.

**Figure 2:**
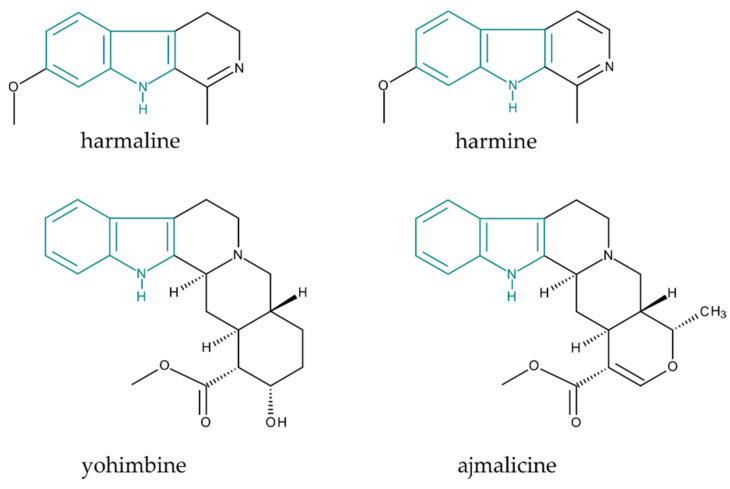
Structure of monoterpenoid indole alkaloids selected for this study (indole ring marked in teal).

Well-known members of this group are psylocin and psilocybin, which are alkaloids isolated from species of the *Psilocybe* genus. The subjects of interest of this study were harmine, harmaline, yohimbine, and ajmalicine (Figure 2), which are compounds belonging to a group of terpene IAs commonly called monoterpenoid indole alkaloids (MIAs); they are formed from tryptamine and the C_10_ terpene unit. It is the largest family of alkaloids in the plant kingdom, consisting of over 2000 compounds of the 10,000 alkaloids identified so far [3]. Many of these natural secondary metabolites are physiologically active in mammals, and they are isolated from various medicinal plants for their specific pharmacological effects.

Among them, harmala alkaloids (isolated for the first time from *Peganum harmala* [4] with harmine, harmaline, harmalol, harmol, and tetrahydroharmine as the most important representatives) are potent inhibitors of monoamine oxidase (MAO, EC 1.4.3.4), i.e., they can decrease the rate of degradation of monoamine neurotransmitters; therefore, they can be used as antidepressants [5]. The other two MIAs included in this study, yohimbine and ajmalicine, are present in many plant species, especially in those of the *Rauvolfia* and *Catharanthus* genera (both of the Apocynaceae family). They grow in tropical and subtropical forests of Asia, Africa, and Latin America, and have been commonly used in the Indian and Western systems of medicine, and in traditional Chinese medicine since antiquity [6,7,8]. The main therapeutic effects of ajmalicine, yohimbine, and other IAs of this type (e.g., reserpine) include antihypertensive and antiarrhythmic properties [9,10]. However, there are also studies where an antitumor effect was demonstrated [11,12].

Although the occurrence of MIAs is relatively well-known, approaches for microscale extraction and sample pretreatment prior to their determination by sensitive analytical method are limited. Similarly, the relative distribution and content of alkaloids in plants are often inconsistent or not well-established [13,14,15]. Consumers in countries where plants rich in MIAs do not grow may receive these plants in the form of dried herbs, extracts, and other preparations that are usually sold as food supplements. In many countries, products of this type are not thoroughly controlled for active and other substances. When we remember the statement of Paracelsus (1493–1541; Swiss alchemist, astrologer, and physician) that ‘the dose makes the poison’ (Sola dosis facit venenum in Latin), i.e., a substance can cause the harmful effect associated with its toxic properties when it reaches a biological system in a certain concentration, we come to the conclusion that these natural products may be dangerous to some individuals. Therefore, the aim of the present research was to establish a fast, reliable, and high-throughput method for the determination of four pharmacologically significant substances from the MIA family for the rapid testing of their content in food supplements of plant origin. The method was validated and subsequently applied for quantitative analysis of these compounds in different, previously unpublished, plant materials. Additionally, part of the study was focused on profiling the selected MIAs in different parts of the tobacco plant to concurrently demonstrate the dependence of alkaloid level on the type of plant tissue (alkaloid distribution across the plant).

## 2. Results

### 2.1. Extraction and Purification Procedure

The extraction and purification procedure was developed with respect to both the relatively hydrophobic (indole ring) and the alkaline character of all studied compounds, which was demonstrated by their dissociation constant pK_a_ ranging from 6.31 (ajmalicine) to 9.55 (harmaline) [16]. Substances of this character were extracted from plant material using 60% MeOH (solvent for relatively hydrophobic substances) containing 0.25% of NH_4_OH (alkaline additive). For the purification of crude extracts containing MIAs, a mixed-mode solid phase extraction (SPE) sorbent Bond Elut Plexa PCX having cation-exchange properties was used to effectively retain MIAs on the sorbent, while substances with acidic properties, and neutral substances were removed from the sample matrix. The ion-exchange group of the used cation-exchange SPE sorbent was bound to a polymer matrix, ensuring its stability in a wide pH range (1–14) and was intended for substances with a dissociation constant pK_a_ of 6–10, which met the range of dissociation constants of the substances of interest. Because the selected extraction solvent contained a base (ammonia), it was necessary to remove it from the sample by evaporation to dryness before applying the sample to the cation-exchange SPE, as the base retention occurs on the cation-exchange resin in an acidic environment where sorbent functional groups are negatively charged, and analytes are positively charged. To induce an acidic environment after evaporation to dryness, a solution containing 60% MeOH with 2% formic acid was selected for the loading of MIA-containing samples. After retention of the basic substances, the hydrophobic interferents were removed from the sorbent by washing with 100% MeOH, while the acidic and neutral substances passed through the column without retention. For the elution of basic substances, including the studied alkaloids, an elution solution containing 60% MeOH with the addition of 5% NH_4_OH was chosen. Using this extraction and purification procedure, the following recovery values for the four standards of monitored alkaloids from the PCX sorbent were found: harmine, 87.86%; harmaline, 51.13%; yohimbine, 80.55%; and ajmalicine, 68.38%.

### 2.2. MIA Analysis by Liquid Chromatography–Mass Spectrometry

**Figure 3:**
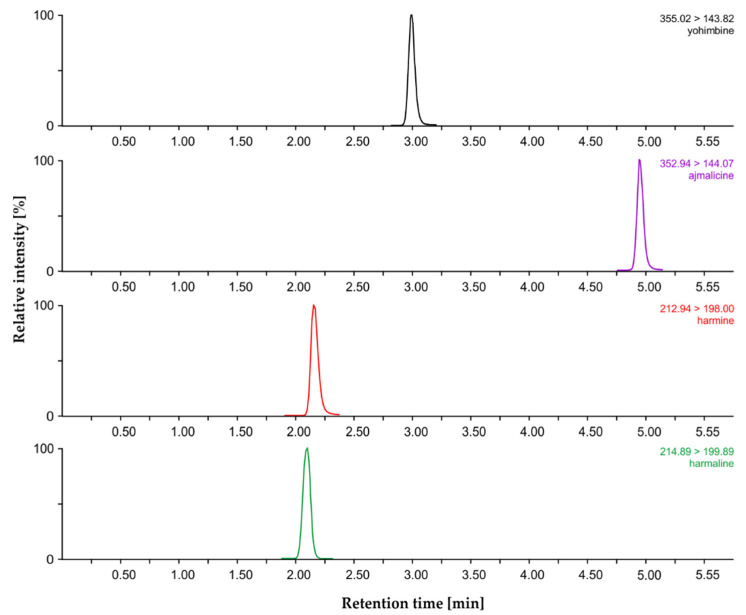
Ultra-high performance liquid chromatography–tandem mass spectrometry (UHPLC–MS/MS) chromatogram of four-indole-alkaloid standard mixture on Acquity UPLC^®^ CSH^™^ Phenyl–Hexyl column using acetonitrile and 10 mM ammonium acetate; pH 10.0.

For the LC separation of the four MIAs of interest, two reversed-phase columns were tested: a ultra-high performance liquid chromatography (UHPLC) column with a sorbent containing a phenyl-hexyl group that selectively retained polyaromatic compounds through π–π interactions (Acquity UPLC^®^ CSH™ Phenyl–Hexyl, 2.1 × 50 mm, 1.7 μm; Waters, Dublin, Ireland), and a UHPLC column with C_18_ particles made by technology offering a superior peak shape for basic compounds under weak ionic strength mobile-phase conditions (Acquity UPLC^®^ CSH^™^, 2.1 × 50 mm, 1.7 μm; Waters, Dublin, Ireland). Ionization efficiency and peak shape were found to be satisfactory when acetonitrile (ACN) (solvent A) and 10 mM ammonium acetate solution were used as components of the mobile phase. Under these conditions, retention times were acceptable and ranged from 2.15 (harmaline) to 5.38 min (ajmalicine) on the CSH column, and from 2.24 to 4.97 min on the phenyl-hexyl CSH column (Figure 3).

However, two important chromatographic characteristics deteriorated when separated on a CSH vs. phenyl-hexyl CSH column. The resolution of the harmine and harmaline critical pair significantly decreased from 90% to 70%, and the average width of all peaks increased by 27% on the CSH column compared to the phenyl-hexyl counterpart (the largest difference in peak width was found for harmaline when it reached 36%). For these reasons, the phenyl-hexyl column was chosen for the further optimization of MIA separation. When optimizing the acidity of the mobile phase, 10 mM ammonium acetate solution adjusted to different pH levels was prepared. At a pH below 8.0 when all analytes were positively charged, significant widening of peak width was observed. Specifically, the width of the harmaline peak was 25% higher when using 10 mM ammonium acetate of pH 7.0 than that of the same mobile phase adjusted to pH 8.6. Moreover, large peak tailing was observed at pH 7.0 to 8.6 for this compound. The symmetry of this peak was achieved by increasing the pH of the mobile phase to a value of 10.0. This behavior might have been related to the dissociation constant of harmaline, which was the highest of the dissociation constants of all monitored substances and reached the value of 9.55 (see above). Therefore, in order to successfully separate such basic substances on this type of stationary phase, it was necessary to adjust the pH of the mobile phase so that the separated substances were in neutral form.

### 2.3. Method Validation

The following performance studies were carried out to prove the suitability of the newly developed method for routine analysis of selected MIAs in biological samples of plant origin: calibration and linearity, limit of detection and limit of quantitation, analytical accuracy, precision and matrix effect.

#### 2.3.1. Method Calibration and Linearity

Calibration curves were created by preparing solutions containing varying amounts of each unlabeled MIA and a known, fixed amount of the methyl-^13^C, D_3_-yohimbine as internal standard (IS) across concentration ranges of 5 to 100 pmol/2 μL of injection. Four separate injections were used to give the resulting calibration curves, which were linear in the selected concentration range for all four MIA compounds investigated (correlation coefficient, *R^2^*, values obtained were in the range of 0.9910 to 0.9985; see Table 1). The linear range for all calibration curves was shown to cover 2 orders of magnitude.

#### 2.3.2. Limit of Detection and Quantitation

The limit of detection (LOD) was evaluated using an approach based on the standard deviation, *s_b_*, of the calibration curve and the slope, and *k*, of a regression curve (LOD = 3 × *s_b_*/*k*). The limit of quantitation (LOQ) was evaluated using a standard deviation/slope ratio approach (LOQ = 10 × *s_b_*/*k*). The LODs and LOQs for MIAs are summarized in Table 1. The lowest LOD/LOQ was found for yohimbine (0.01/0.05 pg), while the most basic harmaline showed the highest values (0.31/1.04 pg).

#### 2.3.3. Analytical Accuracy and Precision

The analytical accuracy of the newly developed UHPLC–(+)electrospray (ESI)–MS/MS method for the determination of selected MIAs was evaluated by the “standard addition method” using two sets of samples: purified extracts of *Nicotiana tabacum* tissue (5 mg fresh weight (FW) of leaf tissue—9th leaf—in 1 mL of extraction solution, six replicates) with the addition of 125 and 250 pmol of authentic MIA standards prior to one step sample purification by SPE. The concentration of each analyte was calculated using the standard isotope dilution method for each plant extract spiked before extraction and compared with the concentration of appropriate standard solution. The analytical accuracy values ranged between 97.7 and 102.0% of the true amount (Table 2). The analytical precision was determined to be in the range of 1.5 to 3.5% (Table 2).

#### 2.3.4. Matrix Effect

The matrix effect (ME) is a known factor in the MS analysis of samples of biological origin, which causes changes in the ionization efficiency of the analyte in the ion source due to the presence of interfering substances originating from the biological matrix of the sample [17]. ME is manifested by the detection of less analytes than is actually the case, which significantly affects the results of quantitative analysis. For this reason, the study of ME was included in this work. ME is of great importance, especially in the determination of substances occurring in trace amounts, but it is also observed for more abundant substances that belong to the group of secondary metabolites of plants, including substances of alkaloid character. The aim of the experiment was to determine the optimal weight of biological material (plant tissue) for the determination of these substances, i.e., to find out what the optimal amount of tissue is leading to the maximal response of individual MIAs in LC–MS analysis that would be affected by interfering substances from the tissue (matrix) to a minimal extent. The comparison of alkaloid and chemical background levels, an experiment with different weights of plant sample, was performed on a commercially available extract of *Pausinystalia yohimbe*, for which the presence of MIAs, especially yohimbine and ajmalicine, is well-described in the literature [18]. Samples were extracted and purified using the one-step SPE-based procedure described in Section 4.3. The presence of yohimbine and ajmalicine was then confirmed by analysis using our newly developed UHPLC–MS/MS method (Section 4.4.), while harmine was also detected; harmaline was not detected in this plant tissue. The highest concentration of all monitored substances was reached by yohimbine, whose levels were found at the level of μg·mg^–1^ dry weight (DW), while the two other alkaloids detected were present at pg·mg^–1^ DW level, i.e., about a thousand times less abundant (Table 3).

**Figure 4:**
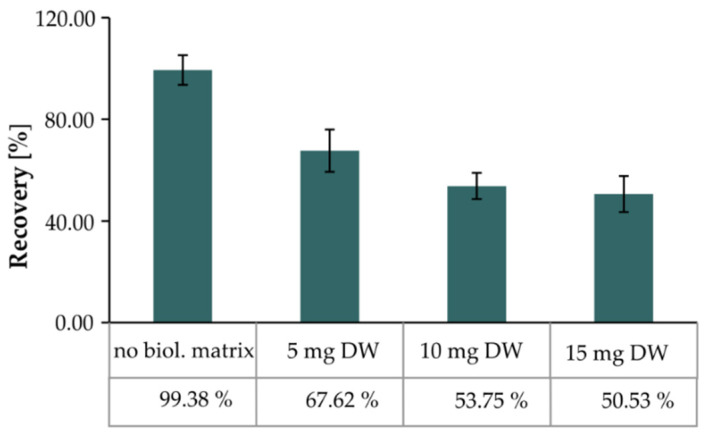
Demonstration of matrix effect—dependence of recovery of methyl-^13^C, D_3_-yohimbine on biological tissue (yohimbe bark) amount. Data/error bars represent mean/standard deviation of three independent determinations.

The effect of the matrix on alkaloid level was observed for harmine and yohimbine, where their amount decreased with increasing sample weight. However, for ajmalicine, the found trend was the opposite (Table 1). The recovery for stable isotopically labelled yohimbine ranged from 67.62% for the 5 mg DW of the biological sample to 50.53% for the 15 mg DW, and clearly represented the ME phenomenon (Figure 4).

Therefore, these experiments showed that the optimal amount of the sample for MIA analysis in yohimbe bark was 1–5 mg DW. As shown in Table 1, using 5 mg sample weight, the content of yohimbine was determined to be 5.29 *±* 0.63 μg·mg^–1^ DW (0.53% *±* 0.06%) in this commercially available sample of yohimbine bark. However, this was not in agreement with the information of the supplier of this product that declared yohimbine content to be 20% (see Section 4.1). In fact, yohimbine content was actually 40 times lower than that stated by the supplier of this food supplement, which might qualify as fraudulent misrepresentation.

The matrix effect can be further presented in the form of a matrix factor (MF), which is a quantity calculated as the ratio of peak area response for each analyte in the presence of the plant matrix ions and peak area response in the absence of matrix multiplied by 100. To study this effect typical for the samples of biological origin, the standard mixture of MIAs (125 pmol each) was added to pure extraction solvent (60% aqueous MeOH, *v/v* containing 0.25% of NH_4_OH) and to the extracts of a fresh tobacco tissue (9th leaf) weighing 5 mg, 10 mg and 15 mg FW after SPE process. Having established a new method for MIA analysis, we tested the extent to which the plant matrix from our samples suppressed the MS signals of interest. The data are summarized in Table 4 and confirm that the strongest matrix effect can be observed for the 15 mg FW (MF mean ∼ 61.1%), while about one-third lower ME was found for 5 mg FW samples (MF ∼ 81.1%),

### 2.4. MIA Determination in Tobbaco

**Figure 5:**
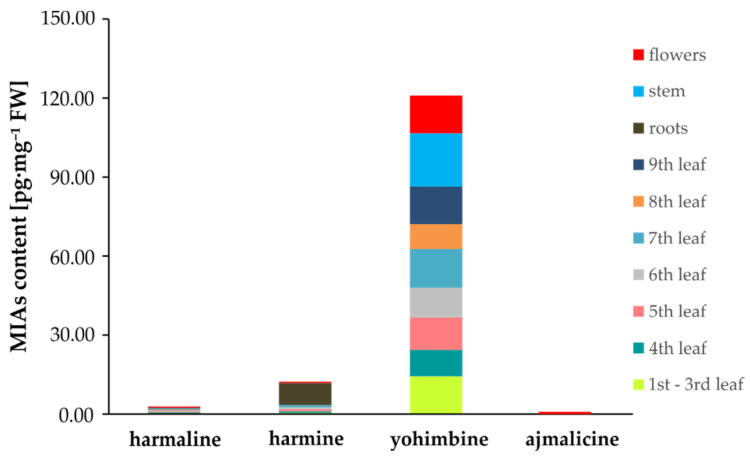
Distribution of individual indole alkaloids in 30-day-old *Nicotiana tabacum* plant. Data/error bars represent mean/standard deviation of three independent determinations.

To accurately determine the distribution of individual substances of interest in different types of tobacco tissue, the seeds of *Nicotiana tabacum*, ecotype SR1 (wild type), were sown and grown for 30 days; after that, the plant was divided into 10 parts according to the scheme described in Section 4.1. Analysis of individual tissue types revealed that the highest content of all four monitored substances was of yohimbine, which represented 89.6% of the total amount of all detected indole alkaloids (Figure 5).

The richest source of yohimbine was found to be a stem of tobacco plant, where its level reached 20.28 pg·mg^–1^ FW (Table 5). Conversely, the poorest sources of this substance were found to be the youngest leaves of the plant (7.76 pg·mg^–1^ FW; Table 5).

**Figure 6:**
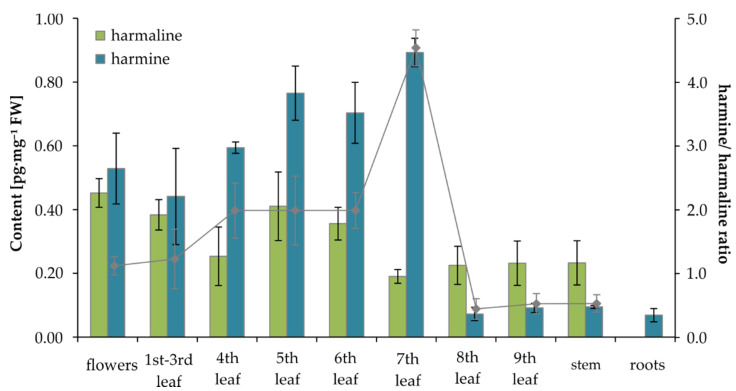
Graph of harmaline and harmine content in different tissue types of 30-day-old *Nicotiana tabacum*. Data/error bars represent mean/standard deviation of three independent determinations.

When we focused on the two structural analogs harmine and harmaline, their total content in this plant was, on average, 1 pg·mg^–1^ FW from flowers to the 7th leaf; then, their total content decreased to 0.32 pg·mg^–1^ FW in the oldest leaves, stems, and roots of the plant, respectively (Figure 6). The lowest levels were found for ajmalicine, which made up only about 1% of the total content of harmine and harmaline. While the content of harmine increased with the age of the plant’s leaves (from the apical part of the plant to the 7th leaf), the trend for harmaline was the opposite, while none was detected in the roots. The ratio of harmine to harmaline basipetally increased from 1.1 in the apical part of the plant to the 7th leaf, where their ratio reached 4.7. In the oldest leaves (8th and 9th) and plant stem, it dropped rapidly to 0.4 (Figure 6).

### 2.5. Determination of MIAs in Tribulus terrestris

*Tribulus terrestris* is a plant in traditional Chinese medicine that is well known for its myriad effects on human health. According to the available literature, it also contains a number of alkaloids, including harmine, harmaline [19], harmane, and norharmane [20]. The content of the two other alkaloids studied in this work was not found in the available literature sources. For quantitation of MIAs using the new UHPLC–MS/MS method, samples of *T. terrestris* were used originating from different countries, and in different compositions of seeds, stems, and other parts of the plant, supplied by Conneco Co., as described in detail in Section 4.1. Analysis showed that the content of harmine and harmaline, when compared to harmane, was at a very low level in the *Tribulus* tissue (Table 6), while harmine was not detected in the *Tribulus* sample originating from Bulgaria and in the sample from China containing only burrs. Furthermore, the *Tribulus* samples were found to contain relatively high levels of yohimbine, and, regardless of the country of origin and composition of the sample, its average content was about 50 pg·mg^–1^ DW, which was the highest level of all indole alkaloids monitored in this type of observed sample. No ajmalicine was detected by this method in *Tribulus* samples.

### 2.6. MIA Determination in Peganum harmala Seeds

As is described in the literature, *Peganum harmala*, similarly to *Tribulus terrestris*, is a very well-known plant in traditional East Asian medicine (eastern Iran, western India). For the purposes of this study, seed samples were commercially obtained (see Section 4.1). Companies that are allowed to supply them to the European Union (EU) market must declare that they are only intended for agricultural purposes, as they cannot be imported as food supplements according to European Union regulations. Reportedly, *P. harmala* seeds contain 2.5% to 4% of a mixture of harmala alkaloids [21], which are characterized by many pharmacological effects, including hallucinogenic effects [22], but also antitumor [23], antimicrobial, or vasorelaxant properties [24]. Analysis using the newly developed UHPLC–MS/MS method revealed that harmala seeds do contain all studied MIAs, with the highest content for harmine (1097 ng·mg^−1^ DW), followed by harmaline (20 ng·mg^−1^ DW), and the lowest for yohimbine and ajmalicine (Table 7).

## 3. Discussion

Alkaloids are the constituents of plant (and, less often, animal or fungal) cells. With regard to their efficient extraction and subsequent precise analysis, it is important to have sound knowledge of the chemical and analytical principles underlying these processes. First, homogenization of plant tissue prior to extraction is necessary for breaking cell walls in tissue [25]. This action allows for the substances present inside the cell to migrate to an appropriate extraction solvent. It is most often completed by grinding or by ultrasonic devices [5,26]. In our method, milligram amounts of plant material were homogenized in plastic safe-locked microtubes with a selected extraction solvent and chemically/mechanically resistant zirconium oxide beads for an appropriate time at a selected frequency. This approach was also successfully applied for sample preparation in ultra-trace analysis of other substances of plant origin, and gives highly repeatable and reproducible results [27,28]. Analyte losses that usually occur during the sample purification procedure can be accounted for by adding internal standards (usually labelled with stable isotopes) to the plant extracts. In addition, this procedure allows a measure of percentage recovery of the target metabolites throughout the purification procedure. Although recovery markers should ideally be included for every analyte that is being quantified, only one internal standard (methyl-^13^C, D_3_-yohimbine) was used for the determination of the four substances in this study. This procedure was chosen because there was a minimal risk of errors in the determination of the three other compounds due to the dissimilarity of their chemical nature. Conditions for the optimal extraction of MIAs were provided by the choice of a solvent with a chemical character similar to the chemical nature of the analyzed substances. Although pure organic solvents such as methanol and ethanol are often commonly used [14,26], the extraction efficiency for compounds with a basic character could be significantly increased by the addition of a basic component to an extraction solution [29]. For this purpose, we added NH_4_OH that met compatibility requirements with MS detection, i.e., it is volatile, does not reduce the signal of the analytes, and does not contaminate the inner part of the quadrupole-based MS analyzer. In order to reduce levels of interfering compounds in highly complex plant extracts, liquid–liquid and/or SPE is a first choice. To ensure high sample throughput and high reproducibility of the method, as well as to reduce the consumption of chemical agents, we used the SPE approach with a sufficient bed size of the mixed-mode sorbent, giving satisfactory recovery and a relatively low volume of cartridge. Moreover, mixed-mode SPE columns packed with a mixture of two types of sorbent allowed us to reduce the number of required purification steps due to more than one separation mechanism that could be exploited using a single column while maintaining high sample-clean-up efficiency. With regard to advanced analytical methods applied for MIA detection and quantitation in samples of plant origin, micellar electrokinetic chromatography (MEKC) [19], high performance liquid chromatography (HPLC) [5,8,15,30], high performance thin layer chromatography (HPTLC) [31], direct analysis in real-time mass spectrometry (DART–MS) [7], and LC–MS/MS [18,26] have been reported to date. Among many available sophisticated instruments, LC–MS/MS offers many advantages in the analysis of these types of compounds mainly due to its separation efficiency and sensitivity, and it was also employed in this study. A comparison of newly developed method parameters with those that were earlier published in regard mainly to the limit of detection and amount of tissue needed for analysis is summarized in Table 8. Unlike commonly used C_8_ [26] or C_18_ [5] stationary phase columns, an LC sorbent modified with phenyl-hexyl groups was shown to be highly efficient for the separation of MIAs as polycyclic compounds.

## 4. Materials and Methods

### 4.1. Plant Materials

**Figure 7:**
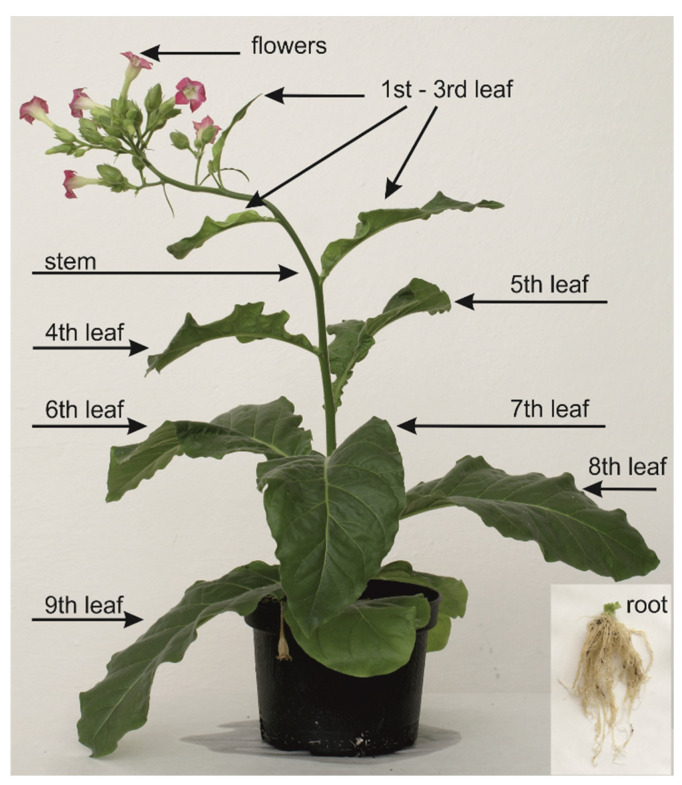
Thirty-day-old tobacco plant. Scheme of plant division into individual parts according to type and age of tissue before extraction and analysis by LC–MS/MS.

**Figure 8:**
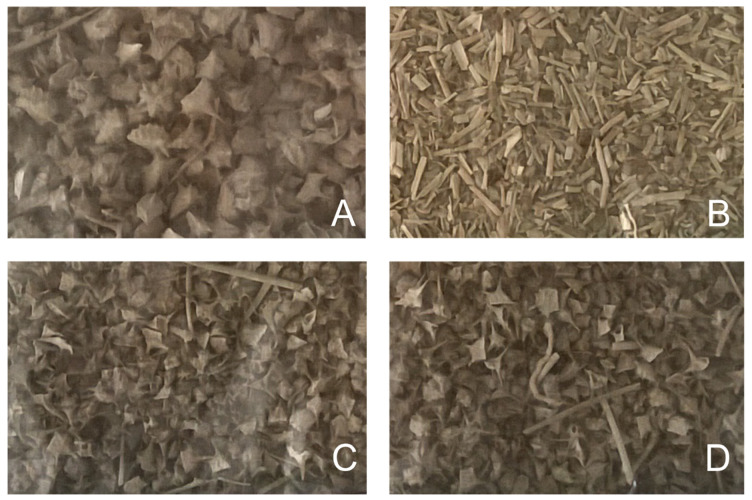
Photo of dry *Tribulus terrestris* tissue provided by Conneco Chemicals Ltd. and used in this study. (**A**) *T. terrestris* burrs including flowers; origin: India. (**B**) Dried whole plant of *T. terrestris* excluding burrs; origin: China. (**C**) *T. terrestris* burrs including stems; origin: Bulgaria. (**D**) Dried whole plant of *T. terrestris* including burrs; origin: China.

**Figure 9:**
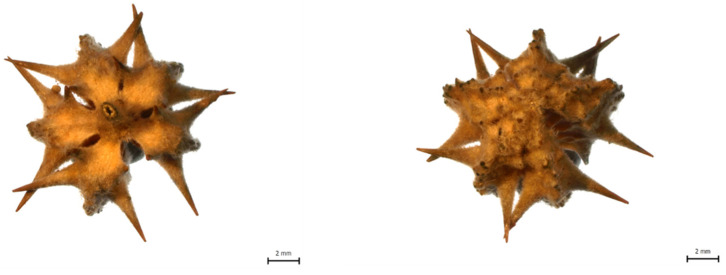
Detailed photo of *Tribulus terrestris* burrs used in this study.

The following plant materials were used for this study: (1) Yohimbe bark extract (*Pausinystalia johimbe*) with declared yohimbine content of 20% (country of origin: not specified, Africa), purchased from Herbal Store, Czech Republic (www.herbal-store.cz). (2) Fresh tobacco tissue (*Nicotiana tabacum* cv. Petit Havana SR1, wild type), with plants grown in the soil of a campus greenhouse of the Centre of the Region Haná for Biotechnological and Agricultural Research, harvested after 30 days of growing under natural conditions, and divided into 10 individual samples, as shown in Figure 7. Samples were immediately frozen in liquid nitrogen and stored in a freezer at –80 °C until extraction and analysis. (3) *Tribulus terrestris*: dry tissue of different types and different country of origin (Figure 8)—burrs (Figure 9) and stems (Bulgaria); stems, whole plants and burrs (China); flowers and burrs (India)—all provided by Conneco Chemicals Ltd. (4) Seeds of *Peganum harmala* (country of origin: Mexico), purchased from Botanico Ltd., Czech Republic (www.botanico.cz).

### 4.2. Chemicals

Indole alkaloids (harmine, CAS 442-51-3; harmaline, CAS 304-21-2; yohimbine, CAS 146-48-5; and ajmalicine, CAS 483-04-5) were obtained from Sigma Aldrich (Steinheim, Germany) at ≥ 95% or higher purity. Stabile isotope-labelled standard of methyl-^13^C, D_3_-yohimbine (CAS 1261254-59-4) was purchased for the purpose of quantitation from Sigma Aldrich, Germany. Formic acid (FA), aqueous ammonia solution (25%, *v/v*), ammonium bicarbonate, ammonium acetate, methanol (MeOH, HPLC grade), and acetonitrile (ACN, HPLC grade) were purchased from Merck (Darmstadt, Germany). Deionized (Milli-Q) water obtained from a Simplicity^®^ UV Water Purification System (Darmstadt, Germany) was used to prepare all aqueous solutions. All other chemicals (analytical grade or higher purity) were from Biosolve Chimie (Dieuze, France).

### 4.3. Extraction and Isolation Procedure

Aliquots of 5 to 15 mg fresh or dry weight (FW, DW) of plant tissue were weighed into 2 mL Eppendorf tubes and 1 mL of 60% MeOH containing 0.25% NH_4_OH as an extraction solution, and 2 mm ceria stabilized zirconium oxide beads (Next Advance Inc., Averill Park, NY, USA) was added for further homogenization using an MM 400 vibration mill at a frequency of 27 Hz for 3 min (Retsch GmbH & Co. KG, Haan, Germany). For quantitative analysis, an internal standard solution containing 125 pmol of methyl-^13^C, D_3_-yohimbine was also added to the samples. Samples were extracted for 20 min in a DT 510 ultrasonic bath (Bandelin GmbH & Co. KG, Berlin, Germany). Homogenates were then centrifuged (36, 670 *g*, 10 min, 4 °C; Beckman Avanti™ 30); supernatants were placed into a clean borosilicate glass test tube and evaporated to dryness at 37 °C using nitrogen evaporator Turbovap^®^ (Biotage, Sweden). After that, the dried sample residues were reconstructed in 60% MeOH containing 2% FA, and subsequently purified using a Visiprep™ solid phase extraction (SPE) vacuum manifold (Supelco^®^, Bellefonte, PA, USA) and mixed-mode polymer-based cation-exchange cartridges (Bond Elut Plexa PCX, 60 mg/3 mL, Agilent Technologies, Santa Clara, CA, USA), activated with 100% MeOH and equilibrated with 60% MeOH + 2% FA before sample loading. After washing the cartridge with 100% MeOH, MIA elution was performed with a solution consisting of 60% MeOH with 5% of NH_4_OH. The elution fraction was evaporated to dryness in vacuo (CentriVap^®^ acid-resistant benchtop concentrator, Labconco Corp., Kansas City, MO, USA).

### 4.4. Indole Alkaloid Analysis by Ultra-High Performance Liquid Chromatography–Tandem Mass Spectrometry (UHPLC–MS/MS)

An Acquity UPLC^TM^ system (Waters, Milford, MA, USA) consisting of binary solvent manager and sample manager modules coupled to a Xevo^®^ TQ MS triple-stage quadrupole mass spectrometer (Waters MS Technologies, Manchester, UK) equipped with an electrospray (ESI) interface and collision cell, Scan Wave, was utilized for MIA quantitation. The entire LC–MS/MS system was controlled by Masslynx™ software (version 4.1, Waters, Manchester, UK). After purification by PCX SPE cartridges, dried samples were reconstituted in 50 μL of 100% MeOH; then, 2 μL of each sample was injected onto a reversed-phase UHPLC column (Acquity CSH™ Phenyl–Hexyl, 2.1 × 50 mm, 1.7 μm, 130 Å; Waters) coupled to the ESI–MS/MS system. MIAs were analyzed in positive ion mode as [M+H]^+^. MIAs of interest were separated (Figure 3) by a linear gradient of ACN (A) and 10 mM ammonium acetate, pH 10.0 adjusted by aqueous ammonia solution (B) at a flow rate of 0.3 mL·min^–1^, 0–1 min, 30:70 (A:B, *v/v*)—15 min, 100:0 (A:B)—15–16 min, 100:0 (A:B). Under these conditions, the substances of interest were eluted within 5 min. Lastly, the column was washed with 100% ACN for 1.5 min and re-equilibrated to initial conditions (30:70 A:B, *v/v*) for 1.5 min. See Table 9 for the retention times of the studied MIAs. The thermostat of the column was programmed to 40 °C, and the temperature inside the autosampler was maintained at 4 °C. Capillary voltage, cone voltage, collision cell energy, and ion source temperatures were optimized for each individual compound using the same setup. Mass spectrometer settings were as follows: capillary voltage, 0.5 kV; cone voltage, 40 V; source temperature, 100 °C; desolvation gas temperature, 350 °C; cone gas flow, 70 L·h^−1^, desolvation gas flow (nitrogen), 500 L·h^−1^; and collision gas flow (argon), 0.15 L h^−1^. The dwell time of each multiple-reaction-monitoring (MRM) channel was calculated to provide 16 scan points per peak with an inter channel delay of 0.1. MS data were recorded in MRM mode (Table 9). All data were processed using MassLynx™ software (ver. 4.1, Waters).

## 5. Conclusions

This work focused on the development of a new microscale method for the extraction, preconcentration, and determination of harmine, harmaline, ajmalicine, and yohimbine, which are indole alkaloids with sympatholytic activity derived from the tryptophan amino acid in plant tissue. We chose 60% MeOH containing 0.25% NH_4_OH as a suitable solvent for the extraction of these substances from plant tissue. Solid phase extraction based on a mixed-mode sorbent with strong cation-exchange properties proved to be suitable for the preconcentration of natural substances exhibiting basic and relatively hydrophobic character. Furthermore, we developed a new and reliable UHPLC–MS/MS-based high-throughput method for profiling the selected indole alkaloids. The applicability of the method was demonstrated for tobacco and *Tribulus terrestris* plant tissue, seeds of *Peganum harmala,* and extract from the bark of *Pausinystalia johimbe*. Experimentally obtained data on the occurrence of individual alkaloids in the given tissue were in agreement with the available literature, except for the presence of ajmalicine and yohimbine in *Tribulus terrestris*, which was detected in this plant species for the first time.

## Figures and Tables

**Table 1:** Validation data for UHPLC–MS/MS analysis of four indole alkaloids.

Compound	Retention Time (min)	Calibration Equation	Correlation Coefficient *R^2^*	LOD (pg)	LOQ (pg)	Dynamic Range (pmol)
Harmaline	2.13 ± 0.01	log y = 0.9707 (log x) − 0.9819	0.9958	0.31	1.04	5–100
Harmine	2.16 ± 0.01	log y = 0.7374 (log x) − 0.2451	0.9935	0.18	0.60	5–100
Yohimbine	2.98 ± 0.01	log y = 1.0147 (log x) − 0.3091	0.9910	0.01	0.05	5–100
Ajmalicine	4.94 ± 0.01	log y = 0.8678 (log x) − 0.2006	0.9985	0.11	0.36	5–100

LOD, limit of detection; LOQ, limit of quantitation.

**Table 2:** Analytical accuracy and precision for determination of monoterpenoid indole alkaloids (MIAs) by the UHPLC–(+) electrospray (ESI)–MS/MS method.

Compound	Determined Spiked Content ^(A)^ (pmol)	Analytical Precision ^(A)^ (%)	Analytical Accuracy ^(A)^ (%)	Determinedspiked Content ^(B)^ (pmol)	Analytical Precision ^(B)^ (%)	Analytical Accuracy ^(B)^ (%)
Harmaline	125.5 ± 0.2	3.5	100.4	252.5 ± 1.4	2.5	101.0
Harmine	123.8 ± 0.9	1.5	99.0	245.5 ± 0.2	2.2	98.2
Yohimbine	125.0 ± 0.3	2.1	100.3	255.1 ± 0.9	2.9	102.0
Ajmalicine	122.5 ± 1.9	2.4	98.0	244.2 ± 1.2	2.3	97.7

(A) Extracts of 5 mg fresh weight (FW) *Nicotiana tabacum* tissue spiked with 125 pmol or 250 pmol (B) of authentic MIA standards, purified by the one solid phase extraction (SPE) step approach and analyzed by UHPLC–MS/MS. The values represent the mean ± standard deviation obtained for six technical replicates prepared and analyzed separately.

**Table 3:** Levels of monitored indole alkaloids in yohimbe bark extract determined by UHPLC–MS/MS.

Sample *W*eight (mg)	Harmaline (pg·mg^–1^)	Harmine (pg·mg^–1^)	Yohimbine (μg·mg^–1^)	Ajmalicine (pg·mg^–1^)
5	ND	32.34 ± 3.69	5.29 ± 0.63	3.46 ± 0.92
10	ND	25.93 ± 2.79	4.01 ± 0.34	7.46 ± 1.24
15	ND	13.59 ± 3.11	3.31 ± 0.37	10.51 ± 1.28

ND, not detected; values represent mean *±* standard deviation of six independent measurements.

**Table 4:** The effect of plant matrix on ionization efficiency of indole alkaloids in fresh tissue of tobacco.

Sample Weight (mg)		Matrix Factor (%)		
	Harmaline	Harmine	Yohimbine	Ajmalicine
5	76.57 ± 0.50	85.55 ± 0.16	94.05 ± 1.34	96.27 ± 1.12
10	69.20 ± 0.33	82.70 ± 0.30	74.74 ± 0.37	69.27 ± 0.50
15	58.68 ± 0.46	70.51 ± 0.21	65.48 ± 1.06	49.75 ± 0.48

The values represent mean *±* standard deviation of six independent measurements.

**Table 5:** Levels of indole alkaloids in 30-day-old *Nicotiana tabacum* plant.

Tissue	Alkaloid Content (pg·mg^–1^)			
	Harmaline	Harmine	Yohimbine	Ajmalicine
Flowers	0.45 ± 0.04	0.53 ± 0.11	14.40 ± 1.68	0.0062 ± 0.0011
1st–3rd leaf	0.38 ± 0.05	0.44 ± 0.15	7.76 ± 1.11	0.0111 ± 0.0010
4th leaf	0.25 ± 0.09	0.59 ± 0.02	10.02 ± 1.36	0.0047 ± 0.0008
5th leaf	0.41 ± 0.11	0.76 ± 0.09	12.38 ± 1.33	0.0135 ± 0.0002
6th leaf	0.36 ± 0.05	0.70 ± 0.10	11.30 ± 1.61	0.0065 ± 0.0015
7th leaf	0.19 ± 0.02	0.89 ± 0.04	14.65 ± 1.09	0.0080 ± 0.0016
8th leaf	0.23 ± 0.06	0.07 ± 0.02	9.53 ± 0.72	0.0067 ± 0.0007
9th leaf	0.23 ± 0.07	0.09 ± 0.01	14.12 ± 1.71	0.0048 ± 0.0011
Stem	0.23 ± 0.07	0.07 ± 0.01	20.28 ± 1.22	0.0090 ± 0.0022
Roots	ND	0.07 ± 0.02	8.04 ± 1.07	0.0056 ± 0.0021

ND, not detected; values represent mean ± standard deviation of three independent measurements.

**Table 6:** Indole alkaloid levels in *Tribulus terrestris*.

Tissue	Alkaloid Content (pg·mg^−1^)			
	Harmaline	Harmine	Yohimbine	Ajmalicine
Burrs + stems (Bulgaria)	0.012 ± 0.005	ND	36.14 ± 2.35	ND
Whole plant (China)	0.004 ± 0.002	1.76 ± 0.18	62.74 ± 5.02	ND
Burrs only (China)	0.004 ± 0.001	ND	43.21 ± 0.77	ND
Flower, burrs (India)	0.005 ± 0.001	0.21 ± 0.05	57.59 ± 3.25	ND

ND, not detected; values represent mean ± standard deviation of three independent measurements.

**Table 7:** Indole alkaloid levels in *Peganum harmala* seeds.

Alkaloid content (pg·mg^–1^)			
Harmaline	Harmine	Yohimbine	Ajmalicine
19.76 ± 0.44	1096.67 ± 14.77	7.25 ± 0.83	0.24 ± 0.03

**Table 8:** The comparison of different methods used for the determination of indole alkaloids.

Instrumental Method Used	Amount of Tissue	Type of Tissue	Limit of Detection	Reference	Capacity of the Method Used
UHPLC–(+)ESI–MS/MS	5 mg	whole plant	0.01–0.31 pg	method presented	4 IAs
HPLC–UV	n.a.	whole plant	0.77–56 mg·g^−1^	[5]	2 IAs
HPLC–UV	0.1 g	roots	6–8 µg·mL^−1^	[8]	3 IAs
HPLTLC	n.a.	seeds	n.a.	[13]	4 IAs
HPLC–UV	1 g	seeds	0.01–0.05 µg·mL^−1^	[14]	2 IAs
HPLC–UV	n.a.	cell culture	n.a.	[15]	22 IAs
UPLC/IM–QTOF–MS *	0.1 g	yohimbe bark	n.a.	[18]	55 IAs
MEKC–UV/LIF	n.a.	none	n.a.	[19]	6 IAs
HPLC–(+)ESI–QTOF–MS/MS	50 g	roots	n.a.	[26]	47 IAs
HPLC–UV–MS	0.5 g	aphrodisiac products	3–60 ng·mL^−1^	[29]	yohimbine
HPLC–UV	2 g	roots	0.05–0.39 µg·mL^−1^	[30]	5 IAs
HPLC–UV	5 g	roots	n.a.	[31]	6 IAs

* ultra-performance liquid chromatography/ion mobility quadrupole time-of-flight mass spectrometry; LIF, laser-induced fluorescence; IAs, indole alkaloids; n.a., not available.

**Table 9:** Optimized MS conditions for detection of each studied indole alkaloid.

Compound	RT (min)	MRM (Q)	Cone Voltage (V)	Collision Energy (V)	MRM (C)	Cone Voltage (V)	Collision Energy (V)
Harmaline	2.13	214.89 > 199.89	40	25	214.89 > 172.11	40	30
Harmine	2.16	212.94 > 198.00	40	25	212.94 > 169.84	40	30
Yohimbine	2.98	355.02 > 143.82	40	30	355.02 > 211.93	40	25
Methyl-^13^C, D_3_-Yohimbine	2.96	359.05 > 143.94	40	30	359.05 > 216.02	40	25
Ajmalicine	4.94	352. 94 > 144.07	40	25	352.94 > 177.91	40	25

RT, retention time; MRM (Q), multiple reaction monitoring transition for compound quantitation; MRM (C), multiple-reaction-monitoring transition for confirmation of compound identity.
